# Design of On-Chip N-Fold Orbital Angular Momentum Multicasting Using V-Shaped Antenna Array

**DOI:** 10.1038/srep09662

**Published:** 2015-05-07

**Authors:** Jing Du, Jian Wang

**Affiliations:** 1 Wuhan National Laboratory for Optoelectronics, School of Optical and Electronic Information, Huazhong University of Science and Technology, Wuhan 430074, Hubei, China

## Abstract

We design a V-shaped antenna array to realize on-chip multicasting from a single Gaussian beam to four orbital angular momentum (OAM) beams. A pattern search assisted iterative (PSI) algorithm is used to design an optimized continuous phase pattern which is further discretized to generate collinearly superimposed multiple OAM beams. Replacing the designed discrete phase pattern with corresponding V-shaped antennas, on-chip N-fold OAM multicasting is achieved. The designed on-chip 4-fold OAM multicasting exploiting V-shaped antenna array shows favorable operation performance with low crosstalk less than -15 dB.

Space-division multiplexing (SDM) with orbital angular momentum (OAM) modes, accompanied by great commitments of increasing system capacity and spectral efficiency considerably, has been witnessed huge progress in mode multiplexing techniques of free-space optical communications and optical fiber link systems^1^,^2^,^3^,^4^. All of those achievements rely on the orthogonality of OAM modes which have helical phase fronts of 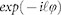 (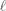 = 0; ±1; ±2; …), where *φ* is the azimuthal angle and 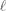 corresponds to the charge value^5^. This orthogonality enables the efficient multiplexing and demultiplexing of spatially superimposed OAM beams with different charge values. Each OAM beam with a specific charge value 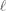 can be seen as an independent channel carrying corresponding data stream. Terabit-scale data transmissions have been realized by introducing the OAM multiplexing technique both in free space and fiber links:1) using eight OAM modes encoded with 16-ary quadrature amplitude modulation (16-QAM) signals to achieve a capacity of 2.56 Tbit/s in free space^1^; 2) using two OAM modes over 10 wavelengths to allow an aggregated rate of 1.6 Tbit/s in 1.1 km fiber^3^.

In addition to multiplexing, multicasting where data on a single channel is duplicated onto multiple channels, has also been desired in an SDM environment from the perspective of efficient optical signal processing in one-to-many communications. By replicating data into orthogonal multiple channels in the optical domain, potential end users are able to acquire the duplicated data speedily and effortlessly. Recently, multicasting in an SDM system based on OAM modes has been reported. 100 Gbit/s (50-Gbaud quadrature phase-shift keying (QPSK)) data channel multicasting from one OAM channel onto multiple OAM channels was demonstrated^6^. It is noted that those multiple OAM channels are all generated by phase-only spatial light modulators (SLM), which are difficult in combination with integrated optical devices due to the large volume of SLM. Very recently, a nanoscale V-shaped antenna meta-surface structure was proposed to achieve the amplitude and phase modulation for cross-polarized scattered fields^7^,^8^. By varying the arm length and the angle between the two arms of V-shaped antenna, one can flexibly adjust the wavefront parameters of scattered fields. This enables the successful generation of single OAM mode, and in principle, also makes it possible to realize on-chip OAM modes multicasting, which however has not yet been reported so far.

In this paper, by combing OAM multicasting together with V-shaped antenna, we design a V-shaped antenna array to realize an on-chip multicasting from a single Gaussian beam to four OAM beams. The crosstalks between multicast OAM channels and their neighboring ones are assessed to be less than −15 dB.

## Results

### Concept of OAM multicasting using V-shaped antenna phase array

**Figure 1:**
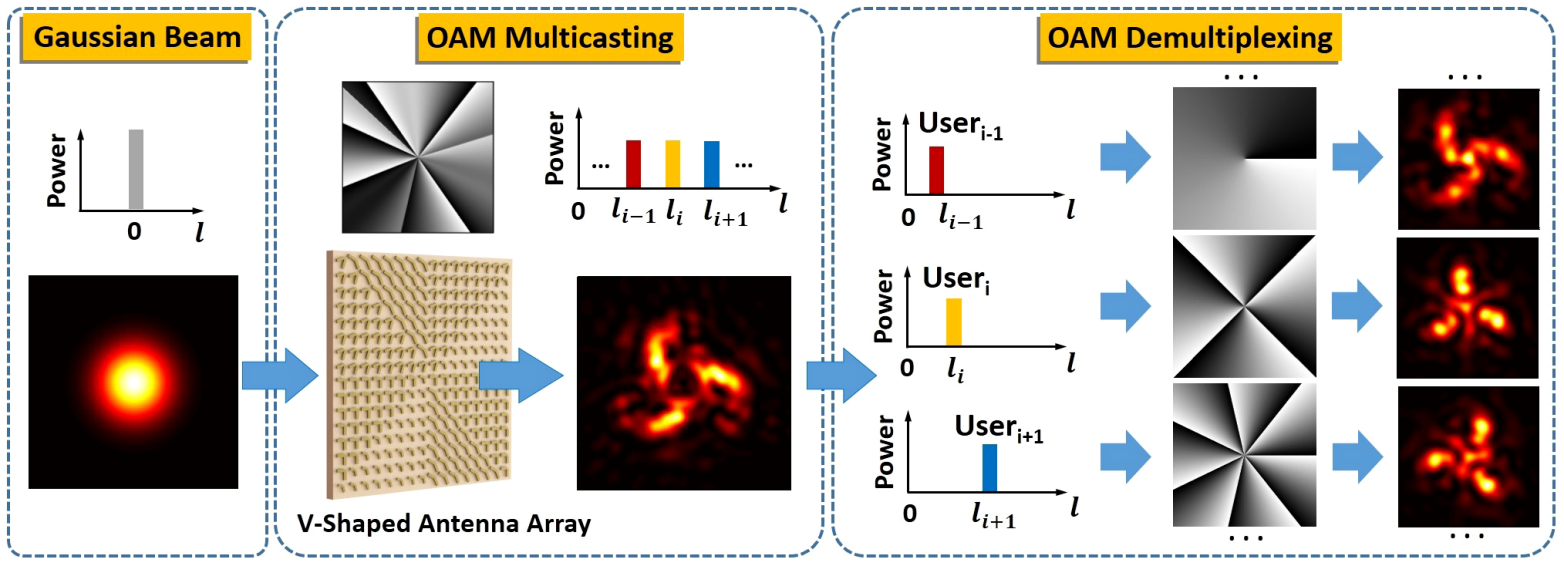
Concept and principle of N-fold multicasting of OAM beams using V-shaped antenna phase array.

Figure 1 displays the concept and principle of N-fold multicasting of OAM beams using V-shaped antennas. At the OAM multicasting side (see Methods), an input Gaussian beam (*l* = 0) with a planar phase front at its beam waist is modulated by a V-shaped antenna array, which is specially designed to be equivalent to a complex multi-OAM phase pattern to generate collinearly superimposed multiple OAM beams. After the modulation, the signal data carried by the input Gaussian beam is duplicated and delivered to N OAM beams which are distinguishable from each other owing to their different charge values. The phase singularity of OAM beams contributes to zero intensity distribution at the centre of superimposed multiple OAM beams. At the OAM demultiplexing side (see Methods), those *N* OAM beams (…, 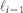, 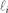, 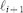, …) are distributed to *N* end users (…, User*_i-1_*, User*_i_*, User*_i+1_*, …), respectively. For each end user, an inverse spiral phase pattern is used to remove the spiral phase front of the desired OAM beam, leading to a bright spot at the centre which can be separated from other OAM beams by spatial filtering. The V-shaped antenna array is the most crucial part in the N-fold OAM multicasting system which directly impacts the quality of multicast OAM beams and the power distribution of multicast OAM channels.

### Characteristics of V-shaped antenna

**Figure 2:**
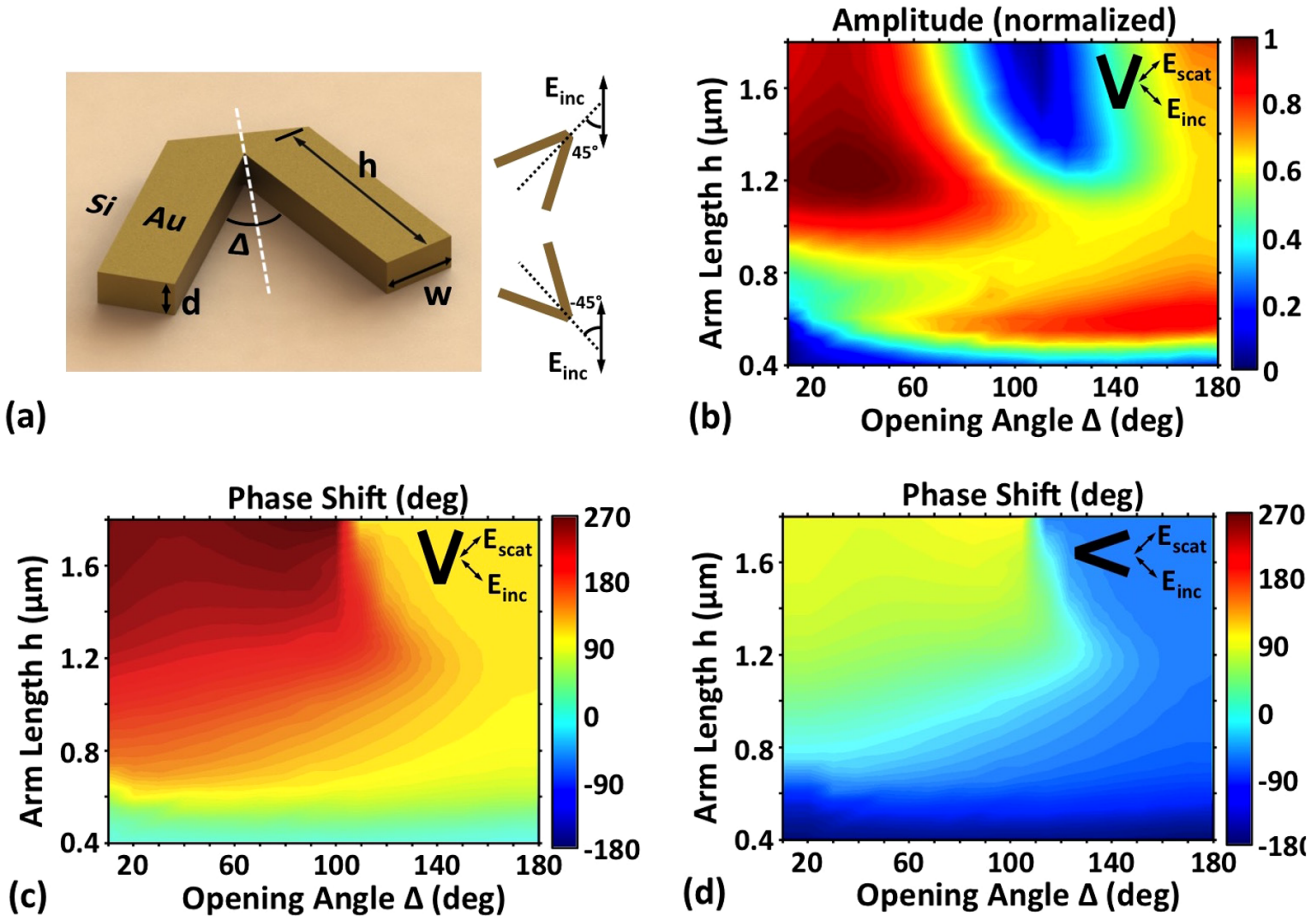
Simulated amplitude and phase shift of the cross-polarized scattered light for V-shaped antenna. (a) Schematic of a V-shaped antenna with d = 50 nm and w = 220 nm. Insets illustrate a V-shaped antenna (the upper one) and its mirror structure (the lower one) with symmetry axis rotation by 90 degree. The polarization of incident light is 45 degree with respect to the symmetry axis of the V-shaped antenna. (b) Amplitude and (c) phase of the cross-polarized scattered light for different antenna geometries (vertical V-shaped antenna with symmetry axis along vertical direction). (d) Phase of the cross-polarized scattered light for different antenna geometries (mirror structure of (c)(d), i.e. horizontal V-shaped antenna with symmetry axis along horizontal direction).

To optimize the V-shaped antenna array for achieving favorable multicasting performance, we first analyze the characteristics of V-shaped antenna. Fig. 2(a) illustrates the schematic of a gold V-shaped antenna which is placed on the surface of silicon substrate. The height (*d*) and width (*w*) of the V-shaped antenna are 50 nm and 220 nm, respectively. The white dotted line indicates the symmetry axis of the V-shaped antenna. The angle between the polarization of incident light and symmetry axis is 45 degree, so both symmetric and antisymmetric modes can be excited in the V-shaped antenna and the scattered light has a substantial component polarized orthogonal to the polarization of the incident light. It is noted that the cross-polarized scattered light (i.e. the polarization of scattered light is orthogonal to the polarization of the incident light) can be easily separated from the incident light simply by use of a polarizer for decoupling. For an existing V-shaped antenna, one can also obtain its mirror structure by rotating 90 degree the V-shaped antenna, i.e. 90 degree rotation for the symmetry axis of the V-shaped antenna. For a typical vertical V-shaped antenna (symmetry axis along vertical direction), by varying the arm length *h* and opening angle *Δ* of the V-shaped antenna, the amplitude and phase shift of the cross-polarized scattered light at an incident wavelength of 8 μm are calculated by the finite-difference time-domain (FDTD) method and plotted in Fig. 2(b) and (c), respectively. One can clearly see from Fig. 2(b) and (c) that the normalized amplitude changes from 0 to 1 and the phase shift varies from 0 to 270 degree. To achieve a full phase shift coverage from 0 to 2π, we also simulate the amplitude and phase of the cross-polarized scattered light for the mirror structure of the vertical V-shaped antenna (i.e. horizontal V-shaped antenna with its symmetry axis along horizontal direction). Similar amplitude of the cross-polarized scattered light is obtained for a V-shaped antenna and its mirror structure, while an additional π phase shift is introduced in the mirror structure, which can be clearly seen in Fig. 2(d). Hence, by combining a V-shaped antenna and its mirror structure with flexible design of geometric parameters, one can achieve easy full phase shift coverage from 0 to 2π. As a consequence, a wide range of amplitude/phase-varied scattered light is achievable, showing the potential to shape or tailor the spatial structure (e.g. wavefront) of the scattered light.

### OAM multicasting using V-shaped antenna phase array

**Figure 3:**
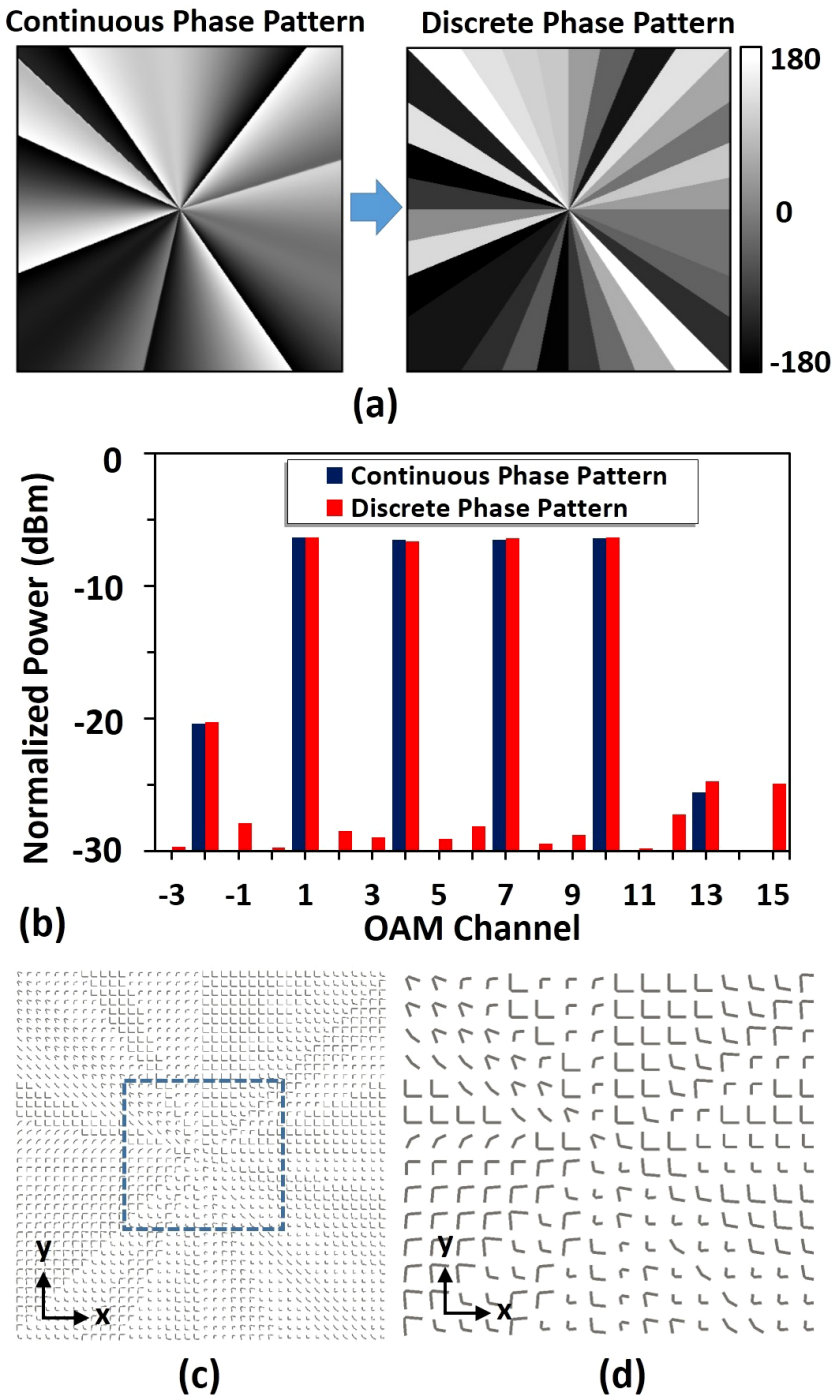
Design of phase pattern. (a) Discretization of ideal continuous phase pattern. (b) Power distributions of OAM channels generated by continuous phase pattern and discrete phase pattern. (c) Top view of designed V-shaped antenna array based on the discrete phase pattern. It repeats with a periodicity of *Γ* = 2 μm in both *x* and *y* directions. (d) Details of designed V-shaped antenna array.

To enable on-chip OAM multicasting, a pattern search assisted iterative (PSI) algorithm (see Methods) is employed to prepare a specific phase pattern for the simultaneous generation of multiple collinearly superimposed OAM beams^9^,^10^,^11^. The left image of Fig. 3(a) displays the calculated phase pattern using PSI algorithm to generate four collinearly superimposed OAM beams with charge values of +1, +4, +7, +10, respectively. One can clearly see from Fig. 3(a) that the phase changes smoothly along the azimuthal direction. However, the calculated continuous phase pattern is not achievable using V-shaped antenna array which can be thought of multiple pixels with discrete phase modulations. In view of the discrete characteristic of V-shaped antenna, it is necessary to replace the calculated continuous phase pattern with a discrete one. The right image of Fig. 3(a) plots the discrete phase pattern which discretizes the continuous phase pattern into 32 values along the azimuthal direction. To evaluate the performance degradation caused by discrete phase pattern on generated collinearly superimposed OAM beams, power distributions of OAM channels (i.e. OAM spectra) generated by both continuous and discrete phase patterns are calculated, compared and depicted in Fig. 3(b). Here we define the crosstalk for all OAM channels by the power ratio of the desired OAM channel (e.g. 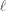 = 4) to its neighboring ones (e.g. 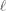 = 3 and 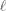 = 5). Slight difference is observed for the target multicast OAM channels when using discrete phase pattern. The crosstalk degradation for all four OAM channels after transmitting through the discrete phase pattern is less than 2 dB. By replacing the discrete phase pattern with corresponding V-shaped antennas, we design the V-shaped antenna array to facilitate multicasting from a single Gaussian beam to four OAM beams as shown in Fig. 3(c)–(d).

**Figure 4:**
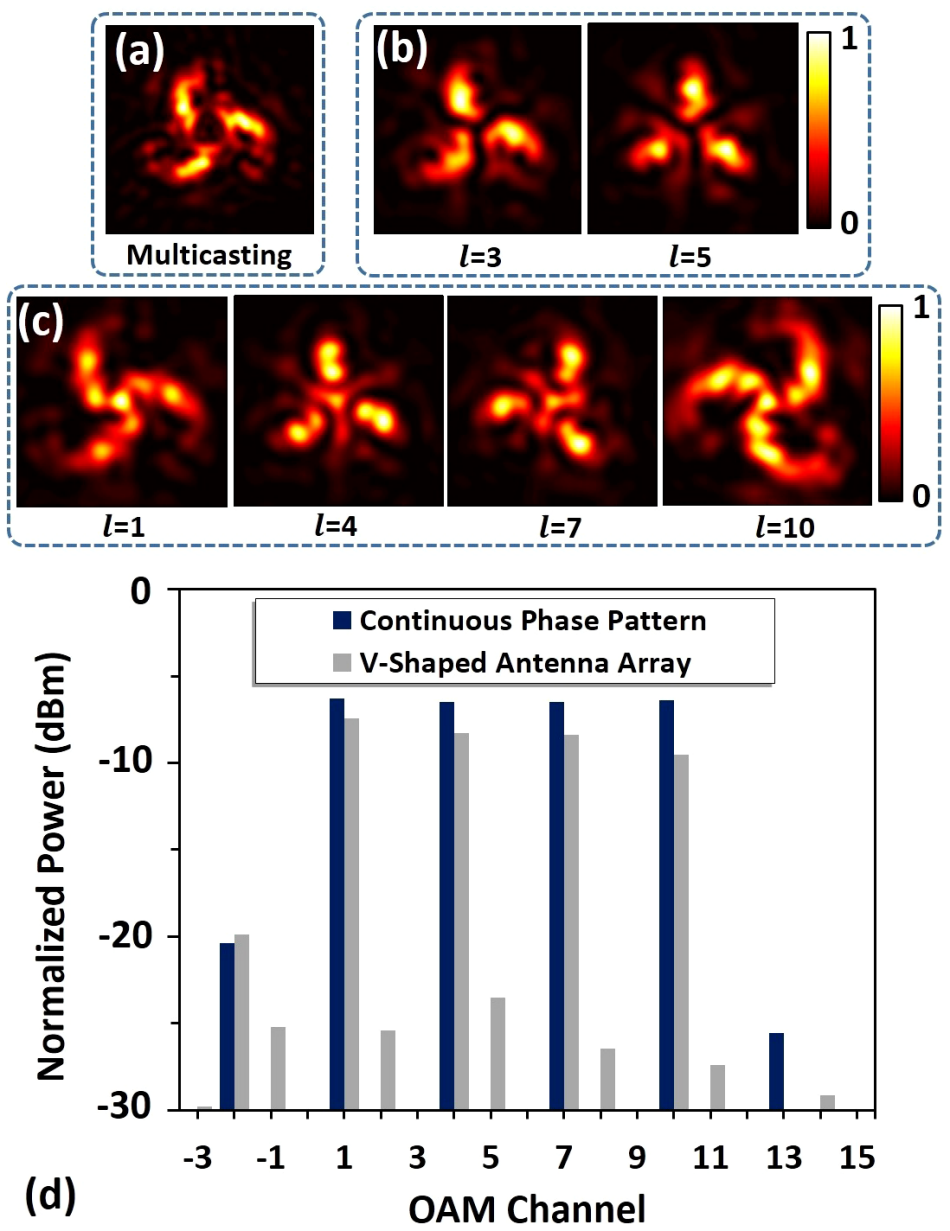
OAM multicasting with four channels using V-shaped antenna phase array. (a) Intensity distribution of collinearly superimposed four OAM beams. (b) Intensity distribution of undesired OAM channels after demultiplexing. (c) Intensity distribution of multicasting OAM channels after demultiplexing. (d) Power distributions of OAM channels generated by the designed V-shaped antenna array and theoretical continuous phase pattern.

Figure 4(a) depicts the calculated far-field intensity distribution of the collinearly superimposed multiple OAM beams generated by the designed V-shaped antenna array which has a triangular dark centre. After demultiplexing, the intensity distributions of OAM channels are shown in Fig. 4(b) and (c). For multicasting OAM channels, there appears a bright spot at the beam centre (Fig. 4(c)), while for undesired channels the beam centre remains a dark region (Fig. 4. (b)). Power distribution of OAM channels generated by the designed V-shaped antenna array is also calculated and displayed in Fig. 4(d). The crosstalks for all multicast OAM channels are less than −15 dB as shown in Fig. 4(d).

## Discussion

In summary, we design a V-shaped antenna array according to the calculated phase pattern to multicast a single Gaussian beam to four OAM beams. PSI algorithm is employed to calculate the optimized continuous phase pattern which is further discretized for easy design of V-shaped antenna array. The OAM multicasting using the designed V-shaped antenna array shows favorable performance with low crosstalk less than −15 dB. However, the proposed V-shaped antenna array for OAM multicasting might suffer relatively low-level energy efficiency, which could be mainly attributed to the weak coupling between the incident and cross-polarized scattered fields and the ohmic losses in metal^12^. There are several possible ways to further improve the energy efficiency: 1) using overlapped electric and magnetic resonances^13^; 2) increasing the thickness of metasurface sheets^14^; 3) combining the metasurface with a reflective ground plane to increase multiple reflections within the film^15^; 4) using dielectric material resonators replacing the metal antenna to decrease the ohmic losses^16^. It is expected that flexible design of V-shaped antenna array may open more interesting applications through the manipulation of spatial structure dimension of light beams.

## Methods

### OAM multicasting and demultiplexing

For the *N*-fold OAM multicasting, when an input Gaussian beam with a planar phase front at its beam waist is spatially modulated by a complex multi-OAM phase pattern to generate *N*-fold collinearly superimposed multiple OAM beams (i.e. OAM multicasting), the complex electric field after OAM multicasting can be expressed as
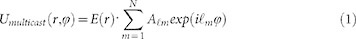
where 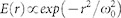 is the complex electric field amplitude at the waist of the Gaussian beam, *r* is the radial distance from the central axis of the Gaussian beam, *ω*_0_ is the waist size, 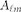 is the weight coefficient of each OAM beam, 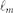 is the charge value of the OAM beam, and *φ* is the azimuthal angle. 
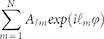
 is the ideal transmission function of *N*-fold OAM multicasting, which will be approximated by a phase-only transmission function *g*(*φ*) = *exp*[i *ψ*(*φ*)] (i.e. complex multi-OAM phase pattern) by adopting a pattern search assisted iterative (PSI) algorithm. Although *N* OAM beams are superposed on one another, each OAM beam is distinguishable from the others due to its unique orbital angular momentum.

For the demultiplexing of multicast OAM beams, an inverse spiral phase mask 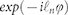 is used to transform the superposed OAM beams as follows
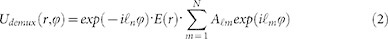


For the desired channel such as user *i* (i.e. one of multicast OAM channels), the corresponding charge value is 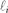 and the inverse spiral phase mask is chosen as 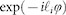. Then the complex electric field of the superposed OAM beams after demultiplexing is written by
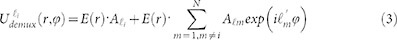
where 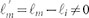. It can be seen that only one of the superposed OAM beams (charge value 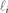) is converted back to a beam with the azimuthal phase term removed. The others are still OAM beams, but with updated charge values from 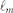 to 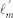. After free-space propagation, the back-converted beam forms a high-intensity bright spot (Gaussian-like) separable from the other OAM beams, which have no intensity at the centre.

For undesired channels (i.e. none of multicast OAM channels with charge value 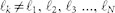), after passing through the corresponding inverse spiral phase mask 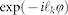, the complex electric field of the superposed OAM beams after demultiplexing is expressed as
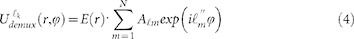
where 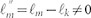. It can be seen that the charge value of each OAM is changed from 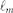 to 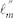 (*m* = 1,2,3...,*N*). However, the total electric field after demultiplexing is still composed of all superposed *N* OAM beams which have no intensity at the centre (dark region).

### Pattern search assisted iterative (PSI) algorithm

Usually, a single OAM beam can be obtained by introducing a phase-only transmission element of 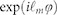 known as circular harmonic^17^ or angular harmonic^18^. The collinearly superimposed multiple OAM beams can be generated if an element comprising multiple circular harmonics is able to be designed. For the simultaneous generation of *N* circular harmonics, the mathematical description of the required transmission function is expressed by
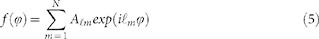
where the complex number 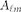 represents the weight coefficient of each circular harmonic (i.e. OAM beam), and 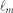 denotes the charge value of the OAM beam. Normally such an *f*(*φ*) is not a phase-only element because it contains both phase and amplitude modulations. In order to simplify the transmission function with both phase and amplitude variations, an approximate phase-only transmission function with constant amplitude would be expected and defined by
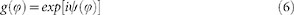

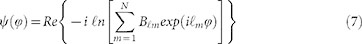


where 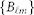 are tentative coefficients for *ψ*(*φ*), and *Re*{} means “real part of”. Discarding the imaginary part in Eq. (7) ensures that *g*(*φ*) is a phase-only transmission function with constant unitary amplitude. *g*(*φ*) can be expanded in Fourier series:
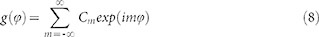


To achieve high performance OAM multicasting, it is desired that the designed phase-only transmission function *g*(*φ*) should approximate the required transmission function *f*(*φ*) as much as possible. In order to evaluate the difference, a parameter of relative root-mean-square error (R-RMSE) is introduced as follows
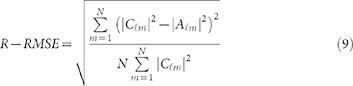


**Figure 5:**
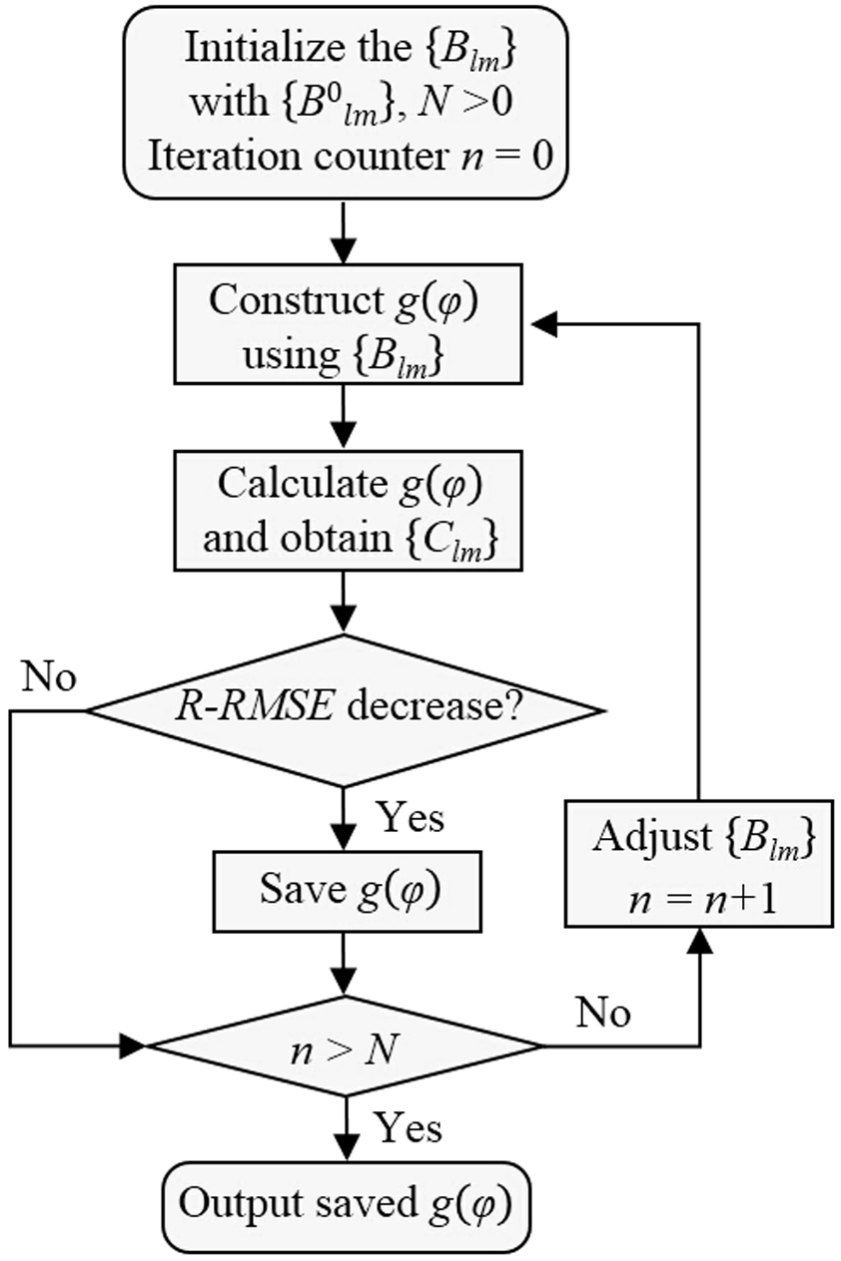
Iterative process of pattern search assisted iterative (PSI) algorithm.

A smaller R-RMSE means a better performance of the designed phase-only element. For given weight coefficients 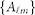 of OAM multicasting, the parameter R-RMSE is determined by 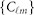 or 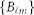. So it becomes a simple minimization problem and one just needs to find suitable 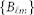 to minimize R-RMSE. A pattern search optimization algorithm is used to estimate the initial value 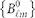 of 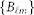
19,20. After getting the initial value of 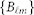, the PSI algorithm with its flow chart shown in Fig. 5 can be adopted to obtain the approximate phase-only transmission function *g*(*φ*).

### Simulation details

We use a three-dimension finite difference time domain (FDTD) method to simulate the spread of electromagnetic field in V-shaped antenna. To analysis the characteristics of V-shaped antenna, a V-shaped cell is simulated. The refractive index of the gold near 8 μm is 8.5 + 46.4i^21^. The interface between silicon and air lies at the centre of the cuboid simulation area, which spans 3 × 3 × 2.5 μm (width × depth × height). Perfectly matched layers (PML) enclose the simulation area in all six surfaces. The mesh cells are 30 × 30 × 10 nm in size in the plane of the antenna and within a 50-nm-thick layer completely encompassing the antenna. Outside this layer, the vertical dimension of the mesh cells is increased to about 150 nm in air and 90 nm in silicon. Then we use a total-field scattered-field (TFSF) plane-wave source encompassing the antenna. The dimensions of the total field region are 3 × 3 × 1.5 μm. The plane wave is launched in the direction perpendicular to the antenna, from the silicon side. A monitor is placed outside this region to isolate the scattered fields. By varying the arm length and opening angle of the V-shaped antenna, the monitor records the amplitude and phase of the scattered field as shown in Fig. 2(b) and (c). To verify the multicasting process, a V-shaped array consists of 400 × 400 cells with a periodicity of Γ = 2 μm is simulated and we use a monitor to record the near-field scattered fields. A near- to far-field transform is then used to calculate the intensity distribution of scattered fields radiated in the direction perpendicular to the plane of the antenna as shown in Fig. 4(a)–(c).

## Author Contributions

J.W. developed the concept and conceived the design. J.D. performed the numerical simulations. J.D. and J.W. analyzed the data. J.D. and J.W. contributed to writing and finalizing the paper. J.W. supervised the project.
